# Heterozygous *fasciated ear* mutations improve yield traits in inbred and hybrid maize lines

**DOI:** 10.1093/plphys/kiae472

**Published:** 2024-09-11

**Authors:** Jinbiao Wang, Qi Zheng, Ruizhong Zhang, Zhaoyu Huang, Qingyu Wu, Lei Liu, Qiang Ning, David Jackson, Fang Xu

**Affiliations:** The Key Laboratory of Plant Development and Environmental Adaptation Biology, Ministry of Education, School of Life Sciences, Shandong University, Qingdao 266237, China; College of Agronomy, Collaborative Innovation Center of Henan Grain Crops, National Key Laboratory of Wheat and Maize Crop Science, Henan Agricultural University, Zhengzhou 450046, China; The Key Laboratory of Plant Development and Environmental Adaptation Biology, Ministry of Education, School of Life Sciences, Shandong University, Qingdao 266237, China; The Key Laboratory of Plant Development and Environmental Adaptation Biology, Ministry of Education, School of Life Sciences, Shandong University, Qingdao 266237, China; Chinese Academy of Agricultural Sciences, Institute of Agricultural Resources and Regional Planning, Beijing 100081, China; National Key Laboratory of Crop Genetic Improvement, Hubei Hongshan Laboratory, Huazhong Agricultural University, Wuhan 430070, China; Hubei Academy of Agricultural Sciences, Food Crops Institute, Wuhan 430064, China; Cold Spring Harbor Laboratory, Cold Spring Harbor, New York, NY 11724, USA; The Key Laboratory of Plant Development and Environmental Adaptation Biology, Ministry of Education, School of Life Sciences, Shandong University, Qingdao 266237, China; Suzhou Research Institute of Shandong University, Suzhou 215123, China

## Abstract

Heterozygous mutations in two genes encoding key regulators of development improve kernel row number in inbred and hybrid maize, revealing their potential for yield improvement.

Dear Editor,

Maize (*Zea mays*) is a major crop worldwide for food, feed and energy. Its ears develop from inflorescence meristems (IM), which give rise a stereotypical series of spikelet pair, spikelet, and floral meristems that form kernels. IM size is associated with kernel row number and kernel number per row, affecting the total kernel number per ear (KNE; Bommert et al. 2013a; Ning et al. 2021). IM activity is orchestrated by the classical CLAVATA (CLV)-WUSCHEL (WUS) regulatory pathway (Wu et al. 2018). In maize, the CLV receptors and ligands include the leucine-rich repeat (LRR) kinase THICK-TASSEL DWARF 1 (TD1) (Bommert et al. 2005) and LRR protein FASCIATED EAR 2 (FEA2) (Taguchi-Shiobara et al. 2001), as well as the two CLAVATA3/EMBRYO SURROUNDING REGION-related (CLE) peptides, ZmCLE7 and ZmFON2-LIKE CLE PROTEIN1 (ZmFCP1) (Je et al. 2016; Rodriguez-Leal et al. 2019). In addition, the G protein α subunit COMPACT PLANT 2 (CT2) (Bommert et al. 2013b) and β subunit Gβ (ZmGB1) (Wu et al. 2020), as well as the pseudokinase CORYNE (ZmCRN) act as downstream signaling components of FEA2 (Je et al. 2018). Mutations in CLV-related genes cause overproliferated IMs, fasciated ears with extreme kernel row number, disorganized kernels, and shorter cobs, ultimately diminishing yield. Manipulating these genes, either by mutations in protein coding or cis-regulatory regions can fine-tune IM activity to increase kernel row number while maintaining normal ear architecture offering possibilities to improve yield (Bommert et al. 2013a; Je et al. 2016; Liu et al. 2021; Li et al. 2022). However, the potential of the null alleles of these genes has been largely overlooked, leading us to ask if they could be used in a dosage specific manner to enhance yield traits in a heterozygous state.

In this study, we scored the kernel row number in heterozygotes of six *fea* mutants, *fea2*, *td1*, *ct2, Zmcle7*, *Zmcrn,* and *Zmgb1,* to investigate whether they have a quantitative impact. These mutants have fasciated ears in B73 inbred, except for *Zmgb1*, which is not viable in B73, and develops fasciated ears when the lethality is suppressed in CML103 (Supplementary Fig. S1;Wu et al. 2020). To control for genetic background effects, each heterozygous *fea* mutant (*fea/+*) was crossed with B73 wild type (WT) and KRN was assessed for heterozygotes and WT siblings in F1. We also scored segregated heterozygotes and WT controls in different hybrids from crosses between heterozygotes in B73 and other backgrounds (Mo17, W22, A619, RP125, KN5585, C7-2, and Z58). Mature ears heterozygous for different mutations in inbred and hybrids had normal ear architecture and kernel row organization similar to WT siblings (Fig. 1A and C, Supplementary Fig. S2A). Strikingly, *Zmcrn* heterozygotes (*Zmcrn/+*) had ∼0.5 to 1.4 more rows than the WT control in B73 inbred and hybrids with data from Sanya (18°N, 108°E; Fig. 1B) and Qingdao (36°N, 120°E; Supplementary Fig. S3A). We also found that *Zmcle7* heterozygotes had increased KRN relative to the controls in B73 inbred and hybrids (Fig. 1D, Supplementary Fig. S3B). In contrast, no significant increase in KRN was observed for *td1*, *gb1*, *ct2*, or *fea2* heterozygotes relative to their WT controls in either inbred or hybrids, except a small increase in *ct2*(B73)/W22 hybrid (Supplementary Fig. S2, B to E). Taken together, our data revealed that *Zmcrn* and *Zmcle7* heterozygotes can quantitatively enhance KRN in both inbred and hybrids, highlighting their potential for enhancing grain yield.

**Figure 1.**
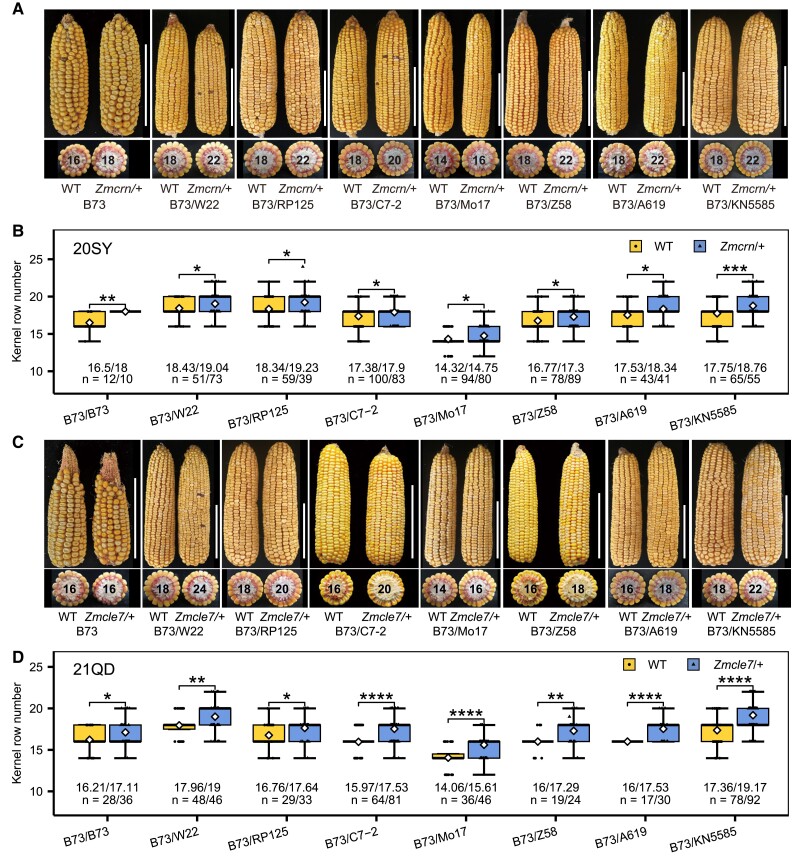
Heterozygosity at *Zmcrn* and *Zmcle7* improve KRN in inbred and hybrid maize lines. **A)** Representative mature ears of WT and *Zmcrn* heterozygotes in B73 inbred and the indicated hybrids, showing lack of ear fasciation. WT, wild type; *Zmcrn/+*, *Zmcrn* heterozygotes. Scale bar: 10 cm. **B)***Zmcrn* heterozygosity significantly increased KRN compared to WT sib controls in B73 inbred and the indicated hybrids. KRN was scored at Sanya in 2020 (20SY). **C)** Representative mature ears of WT and *Zmcle7* heterozygotes in B73 inbred and the indicated hybrids, showing lack of ear fasciation. WT, wild type; *Zmcle7/+*, *Zmcle7* heterozygotes. Scale bar: 10 cm. **D)***Zmcle7* heterozygosity significantly increased KRN compared to WT sib controls in B73 inbred and the indicated hybrids. KRN was scored at Qingdao in 2021 (21QD). For **B)** and **D)**, data are presented as box plots with two-tailed Student's *t*-test. * *P*-value ≤ 0.05, ** *P*-value ≤ 0.01, *** *P*-value ≤ 0.001. **** *P*-value ≤ 0.0001. The box indicates the first or third quartile with a median, whiskers further extend by ±1.5 times the interquartile range from the limits of each box, and the white diamond represents the mean. The mean values and the number of plants (*n*) used for the statistical analysis are listed. The source data can be found in Supplementary Tables S1 and S2.

To further evaluate the impact of *Zmcrn* heterozygosity on grain production, we scored additional yield related traits including grain yield per ear (GYE), ear weight (EW), KNE, ear diameter (ED), kernel depth (KD), ear length (EL), kernel numbers per row (KNR), and hundred-kernel weight (HKW) in different hybrids. Remarkably, *Zmcrn* heterozygotes increased GYE by 4% to 9% in three hybrids: *Zmcrn* (B73)/C7–2, *Zmcrn* (B73)/W22, and *Zmcrn* (B73)/RP125, with data from two seasons (Fig. 2A, Supplementary Fig. S4A). *Zmcrn* heterozygotes also had increases in EW in these three hybrids (Fig. 2B, Supplementary Fig. S4B). The rest traits including KNE, ED, KD, EL, KNR, and HKW were either increased or unaffected (Fig. 2C–H, Supplementary Fig. S4C-, S to H). In four other hybrids: *Zmcrn* (B73)/KN5585, *Zmcrn* (B73)/Mo17, *Zmcrn* (B73)/Z58, and *Zmcrn* (B73)/A619, there was no significant increase in GYE and EW (Supplementary Fig. S5A and B) and no significant effect or minor effect on the other traits (Supplementary Fig. S5C and H). Our data suggest that *ZmCRN* is a promising locus for improving yield traits, though its performance varies across different genetic backgrounds, likely due to complex trait interactions and variations in heterosis. In addition, a candidate gene association study in a maize panel of 507 inbred lines found that *ZmCRN* is significantly associated with KRN (Supplementary Fig. S6). Lines with the favorable haplotype had higher KRN (Supplementary Fig. S6B) and this haplotype was positively selected during domestication (Supplementary Fig. S6C). Taken together, our data revealed that natural variation in *ZmCRN* is associated with KRN, and *ZmCRN* is a promising locus for breeding high-yielding varieties.

**Figure 2.**
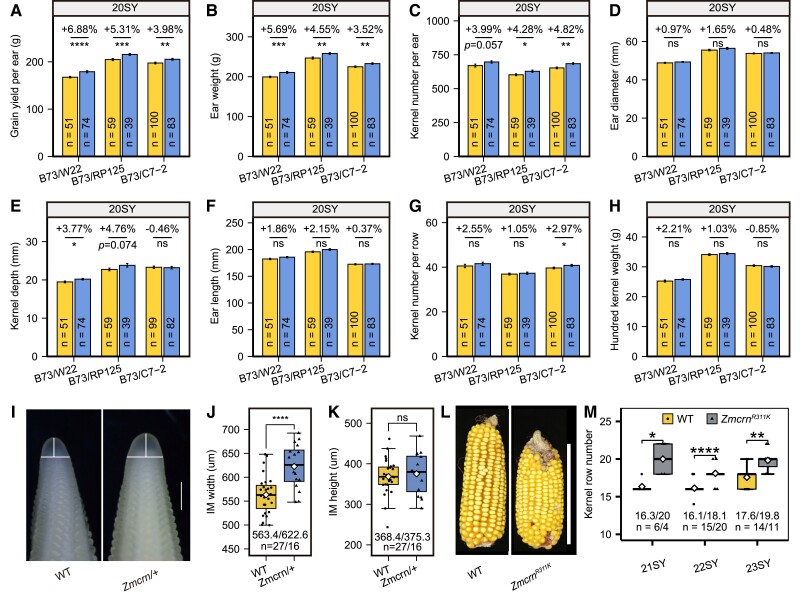
Heterozygosity at *Zmcrn* improves GYE and EW in hybrid maize lines and weak alleles of *Zmcrn* enhance KRN. **A**–**H)** Scoring of eight yield traits including GYE A), EW B), KNE C), ED D), KD E), EL F), kernel number per row G) and HKW H) for segregated *Zmcrn*/+ and WT in B73/W22, B73/RP125, and B73/C7-2 hybrids. All yield-related trait scoring were performed at Sanya in 2020 (20SY). Data are presented as mean values ± SE, * *P*-value ≤ 0.05, ** *P*-value ≤ 0.01, *** *P*-value ≤ 0.001, **** *P*-value ≤ 0.0001, with two-tailed Student's *t*-test. ns indicates nonsignificant. Yellow bars: WT. Blue bars: *Zmcrn/+*. The source data can be found in Supplementary Table S4. **I)** Microscopy images showing representative ear primordia of WT and *Zmcrn/+.* IM width and height are indicated by white lines. IM: Inflorescence meristem. Scale bar: 500 μm. The scale bar applies to both WT and *Zmcrn/+*. **J) and K)** IM diameters of WT and *Zmcrn/+* revealed wider IMs in *Zmcrn/+* compared to the control, while IM heights of WT and *Zmcrn/+* show no significant difference. Data are presented by box blots with two-tailed Student's *t*-test. *** *P*-value ≤ 0.001. The box indicates the first or third quartile with a median, whiskers further extend by ±1.5 times the interquartile range from the limits of each box, and the white diamond represents the mean. The source data can be found in Supplementary Table S5. **L)** Representative mature ears of WT and *Zmcrn^R311K^,* showing nonfasciated ears. Scale bar: 10 cm. The scale bar applies to both WT and *Zmcrn^R311K^.***M)***Zmcrn^R311K^* increased KRN relative to the WT siblings with data collected from at Sanya in 2021, 2022, and 2023 (21SY, 22SY, and 23SY). Data analysis and *P-*value calculation were performed as described in Fig. 1B. Box plots are defined as in J) and K). The source data can be found in Supplementary Table S6.

To better understand the underlying cause of the increase in KRN in *Zmcrn* heterozygotes, we measured IMs in the B73 inbred (Fig. 2I). We found that *Zmcrn* heterozygotes had significantly wider IMs compared to their WT siblings but unaffected IM height (Fig. 2J and K). Our results suggest that *Zmcrn* heterozygotes have higher meristem activity, leading to the increase in KRN.

To mine additional *ZmCRN* alleles for potential grain improvement, we scored 14 nonsynonymous *Zmcrn* alleles from an EMS mutant library (Supplementary Fig. S7A;Lu et al. 2018). Unlike the *Zmcrn* null mutants, none of these alleles had fasciated ears (Fig. 2L, Supplementary Fig. S7B). Three alleles (*Zmcrn^S266F^*, *Zmcrn^R311K^*, and Zmcrn^S340L^) increased KRN with normal ear architectures, indicating they are weak alleles potentially useful for yield improvement (Fig. 2M, Supplementary Fig. S7C). One allele (*Zmcrn^P350s^*) decreased KRN, suggesting it was a hypermorph (Supplementary Fig. S7C). No significant difference in KRN was detected for the other EMS alleles. ZmCRN was previously characterized as pseudokinase lacking the conserved feature of a typical kinase (Nimchuk et al. 2011). Interestingly, all four alleles causing a difference in KRN were located within its pseudokinase domain, indicating a crucial nonkinase function. These variations were not found in the maize association panel of 507 inbred lines, which is in line with the fact that no natural variations at coding region of *ZmCRN* were identified in the association analysis. Our results suggest that induced variations through EMS mutagenesis or CRISPR base editing could enhance yield traits with more variations than found in nature.

Studies on CLV-related mutants in maize have advanced our fundamental understanding on meristem development. However, null alleles of these genes often have severe phenotypes that affect yield. The fasciated ear phenotype appeared to be a recessive trait, as heterozygotes for the six null mutants have normal ear architecture, both in inbred or hybrids. However, we found that *Zmcrn* and *Zmcle7* heterozygotes had quantitative effects on increasing KRN in inbred and hybrids. In contrast, heterozygotes for the other four mutants showed no obvious effects on KRN. In all heterozygous *fea* mutants, the normal transcript levels were reduced to approximately half of that in WT siblings (Supplementary Fig. S8), but only *Zmcrn* and *Zmcle7* heterozygotes significantly increase KRN. This suggests that *ZmCRN* and *ZmCLE7* are more sensitive to dosage change than other *FEA* genes, and are more promising targets for gene manipulation to improve yield traits such as KRN. Future large-scale yield tests with commercial planting conditions and additional environments will better reflect the effects of *Zmcrn* and *Zmcle7* heterozygotes on improving yield traits (Khaipho-Burch et al. 2023). *ZmCRN* and *ZmCLE7* have the lowest levels in developing ear primordia among the *fea* genes (Supplementary Fig. S9), which provides a possible explanation why these two genes are more sensitive to dosage change. Besides, the haplotype variation associated with KRN laying in the 3′UTR region of *ZmCRN* likely impacts transcript levels, as polymorphisms in 3′UTR regions can cause variation in gene expression levels or mRNA stability (Wang et al. 2021, 2024), which is also in line with the fact that *ZmCRN* is sensitive to dosage. Our results reveal that classical null mutants with qualitative phenotypes can have quantitative effects on important traits. Such effects have typically been observed in alleles with variations in cis-regulatory elements.

## Supplementary Material

kiae472_Supplementary_Data

## Supplementary data

The following materials are available in the online version of this article.

**Supplementary Figure S1.** Ear phenotype of different *fea* mutants.

**Supplementary Figure S2.** The KRN is not significantly affected by *td1/+*, *fea2/+*, *ct2/+* and *gb1/+* compared to their WT control in the B73 inbred and indicated hybrids.

**Supplementary Figure S3.** Heterozygosity at *Zmcrn* and *Zmcle7* improves KRN in inbred and hybrid maize lines.

**Supplementary Figure S4.** Heterozygosity at *Zmcrn* improves grain yield per ear and ear weight in B73/W22, B73/RP125 and B73/C7-2 hybrids at Qingdao in 2020.

**Supplementary Figure S5.** Scoring of eight yield traits for *Zmcrn* heterozygotes and WT in B73/Mo17, B73/Z58, B73/A619 and B73/KN5585 hybrids at Qingdao and Sanya in 2020.

**Supplementary Figure S6.** Association analysis of *ZmCRN* with KRN.

**Supplementary Figure S7.** Identification and ear scoring of nonsynonymous alleles of *Zmcrn*.

**Supplementary Figure S8.** Transcript levels of *FEA* genes in WT, heterozygotes and homozygotes by RT-qPCR assay.

**Supplementary Figure S9.** FPKM values for different *FEA* genes in ear primordia at various developmental stages.

**Supplementary Table S1.** The KRN of *Zmcrn* heterozygotes and WT in B73 inbred and different hybrid backgrounds.

**Supplementary Table S2.** The KRN of *Zmcle7* heterozygotes and WT in B73 inbred and different hybrid backgrounds.

**Supplementary Table S3.** The KRN of *td1/+*, *fea2/+*, *ct2/+*, *gb1/+* and their WT control in B73 inbred and different hybrid backgrounds.

**Supplementary Table S4.** Performance of yield-related traits in *Zmcrn* heterozygotes and WT in different hybrid backgrounds.

**Supplementary Table S5.** The IM size of *Zmcrn* heterozygotes and WT in B73 inbred background.

**Supplementary Table S6.** The KRN of nonsynonymous alleles of *Zmcrn*.

**Supplementary Table S7.** Primers used in this study.

## Dive Curated Terms

The following phenotypic, genotypic, and functional terms are of significance to the work described in this paper:

WUS Gramene: AT2G17950
WUS Araport: AT2G17950
