# Supplementary material for: Heterozygous fasciated ear mutations improve yield traits in inbred and hybrid maize lines
Source: Plant Physiol. 2024 Sep 11;196(4):2291–5. doi: 10.1093/plphys/kiae472 (PMC11637988; doi:10.1093/plphys/kiae472)
Supplement: kiae472_Supplementary_Data [file kiae472_supplementary_data.zip › Supplementary Data.pdf]

Supplementary Figure S1

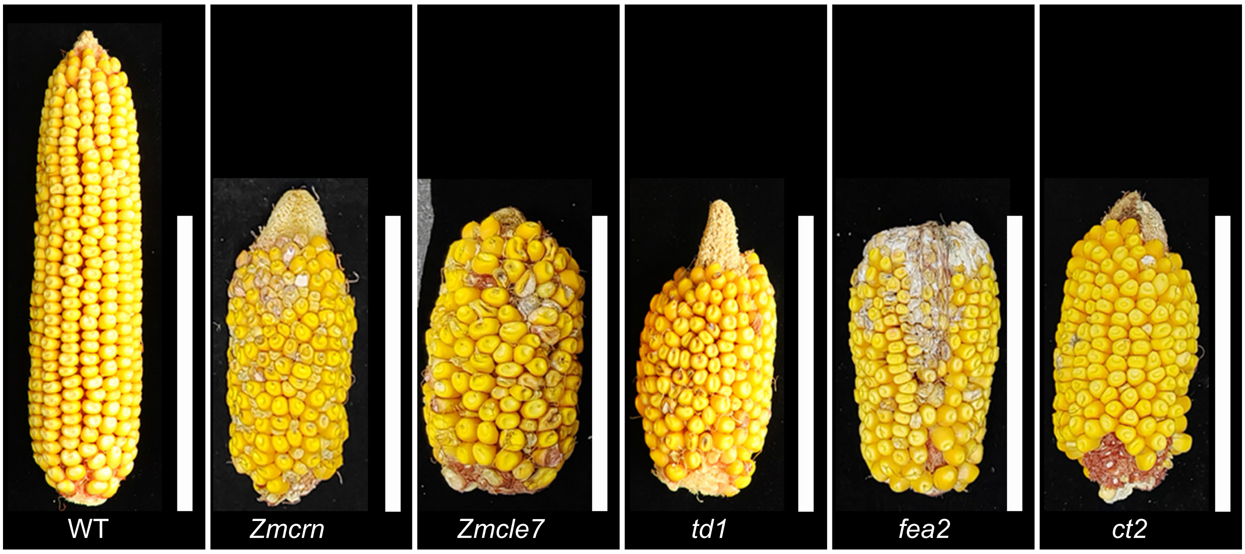

Supplementary Figure S1. Ear phenotype for different *fea* mutant. Representative mature ears of *fea* null alleles in B73 inbred showed fasciated ears. Scale bar: 10 cm

Supplementary Figure S2

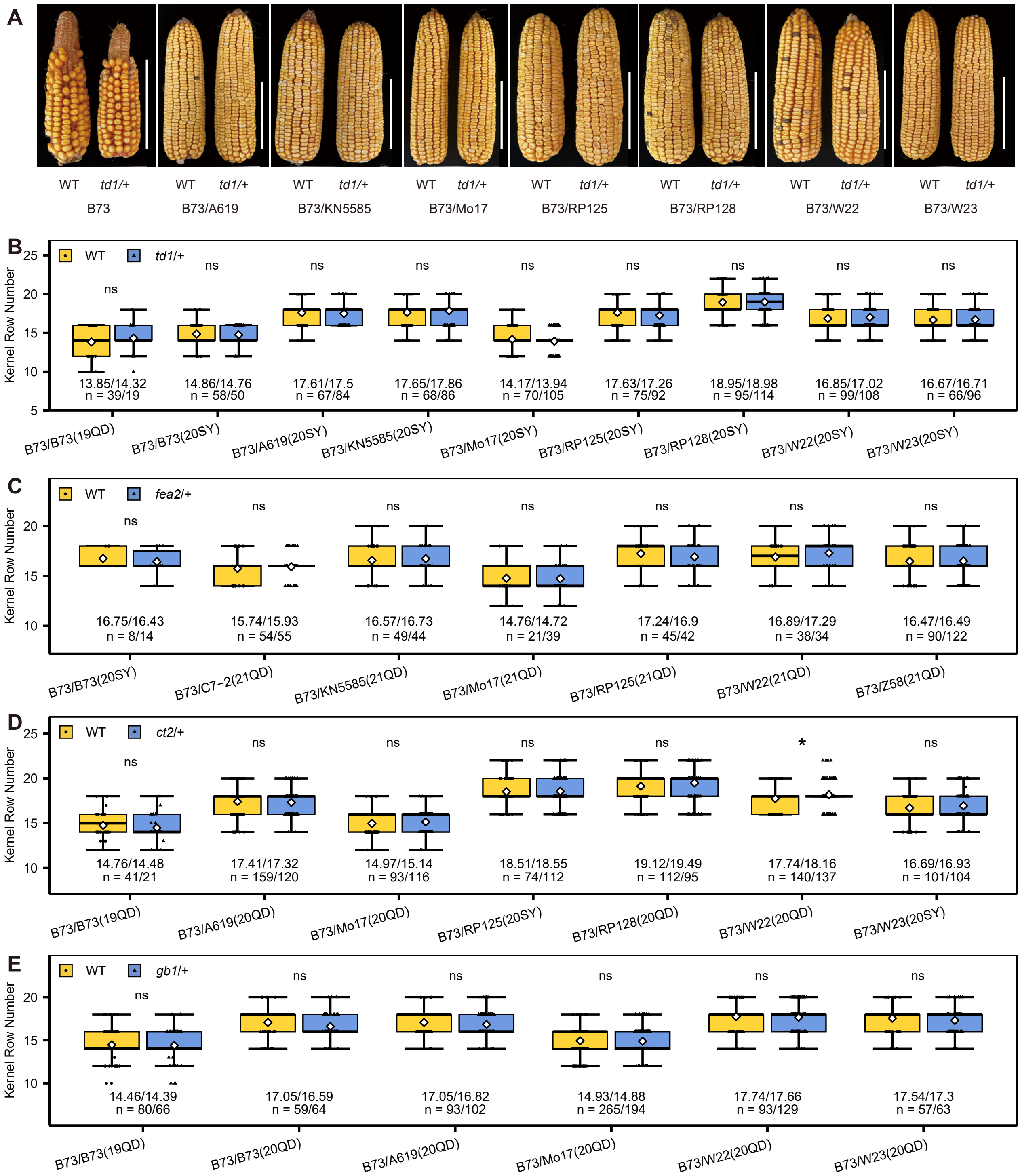

Supplementary Figure S2. The KRN is not significantly affected by *td1/+*, *fea2/+*, *ct2/+* and *gb1/+* comparing to their WT sibling control in B73 inbred and the indicated hybrids. A) Representative mature ears of WT and *td1* heterozygotes in B73 inbred and the indicated hybrids, showing non-fasciated ear. Images were digitally extracted for comparison. Scale bar: 10 cm. **B-E)** The scoring of KRN for *td1/+* **B)**, *fea2/+* **C)**, *ct2/+* **D)** and *gb1/+* **E)** in the tested hybrids showed no significant difference in most comparisons. KRN scoring were performed at either Qingdao in 2019, 2020 and 2021 (labeled as 19QD, 20QD and 21QD) or Sanya in 2020 (20SY). Data are shown by box blots with two-tailed Student's *t*-test. * *p*-value ≤ 0.05. The box indicates the first or third quartile with a median, whiskers further extend by ±1.5 times the interquartile range from the limits of each box, and the white diamond represents the mean. The mean values and the number of plants (n) used for the statistical analysis are listed. The source data and *p*-values for are listed in Supplementary Table S3.

Supplementary Figure S3

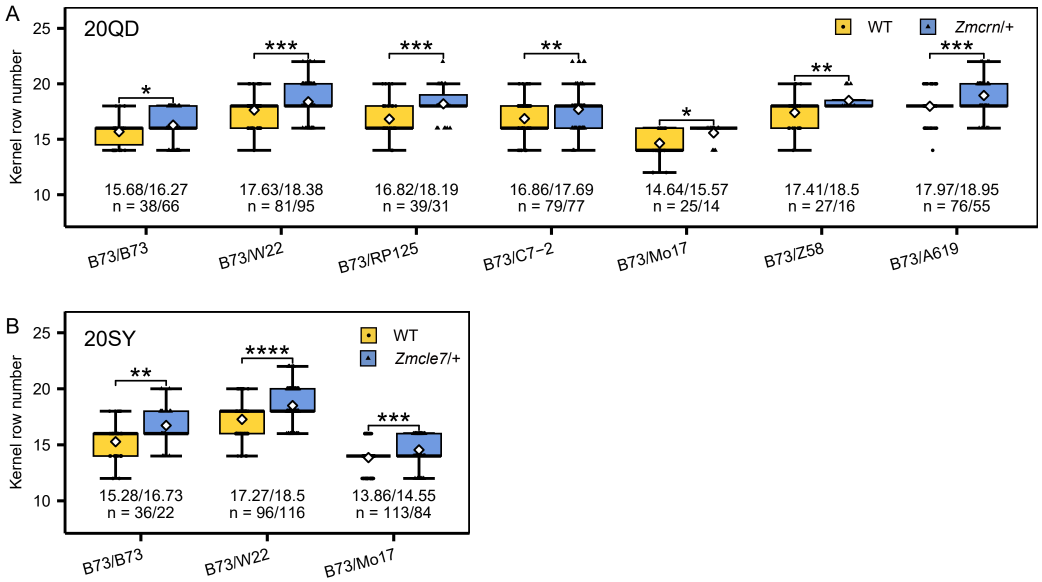

Supplementary Figure S3. Heterozygosity at *Zmcrn* and *Zmcle7* improve KRN in inbred and hybrid maize lines. A) *Zmcrn* heterozygosity significantly increased KRN compared to WT sib controls in B73 inbred and the indicated hybrids. KRN was scored at Qingdao in 2020 (20QD). Data are presented by box plots with two-tailed Student's *t*-test. * *p*-value ≤ 0.05, ** *p*-value ≤ 0.01, *** *p*-value ≤ 0.001, **** *p*-value ≤ 0.0001. The mean values as well as the number of plants (n) used for the statistical analysis are listed. The source data can be found in Supplementary Table S1. **B)** *Zmcle7* heterozygosity significantly increased KRN compared to WT sib controls in B73 inbred and the indicated hybrids. KRN was scored at Sanya in 2020 (20SY). Data analysis and *p*-value calculation were performed as described in Supplementary Figure S3A. The box indicates the first or third quartile with a median, whiskers further extend by ±1.5 times the interquartile range from the limits of each box, and the white diamond represents the mean. The source data can be found in Supplementary Table S2.

Supplementary Figure S4

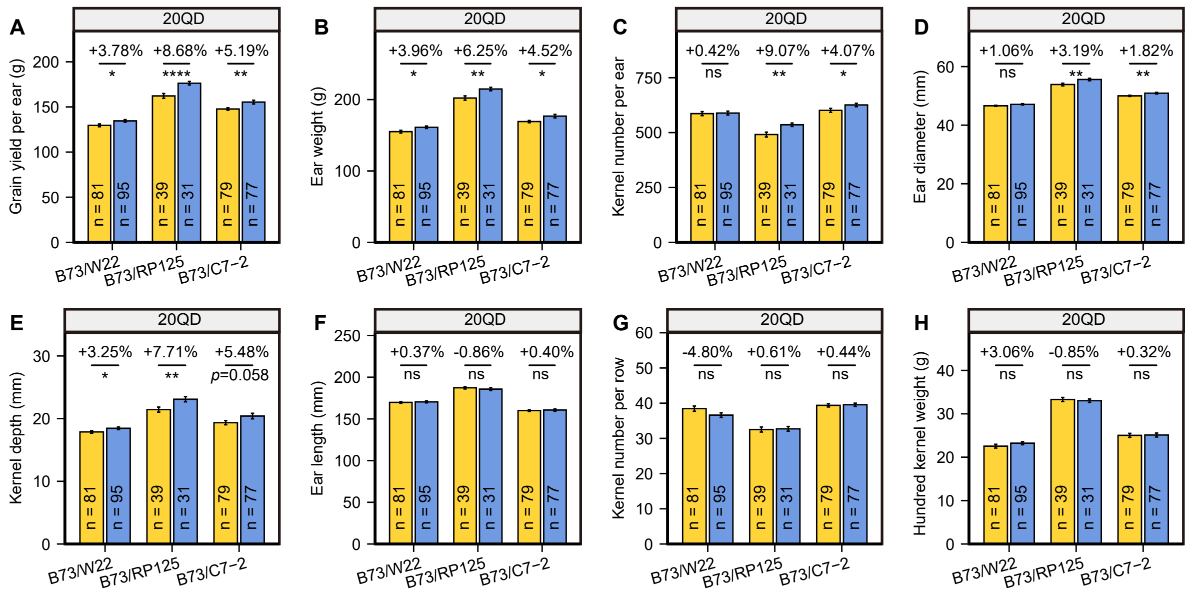

Supplementary Figure S4. Heterozygosity at *Zmcrn* improve grain yield per ear and ear weight in B73/W22, B73/RP125 and B73/C7-2 hybrids at Qingdao in 2020. Scoring of eight yield traits including grain yield per ear **A)**, ear weights **B)**, kernel numbers per ear **C)**, ear diameters **D)**, kernel depths **E)**, ear lengths **F)**, kernel numbers per row **G)**, hundred-kernel weight **H)** for segregated *Zmcrn*/+ and WT in B73/W22, B73/RP125 and B73/C7-2 hybrids. All yield-related trait scoring were performed in 2020 at Qingdao (20QD). Data are presented as mean values ± s.e, * *p*-value ≤ 0.05, ** *p*-value≤ 0.01, *** *p*-value ≤ 0.001, **** *p*-value ≤ 0.0001, with two-tailed Student's *t*-test. ns: not significant. The source data are listed in Supplementary Table S4.

Supplementary figure S5

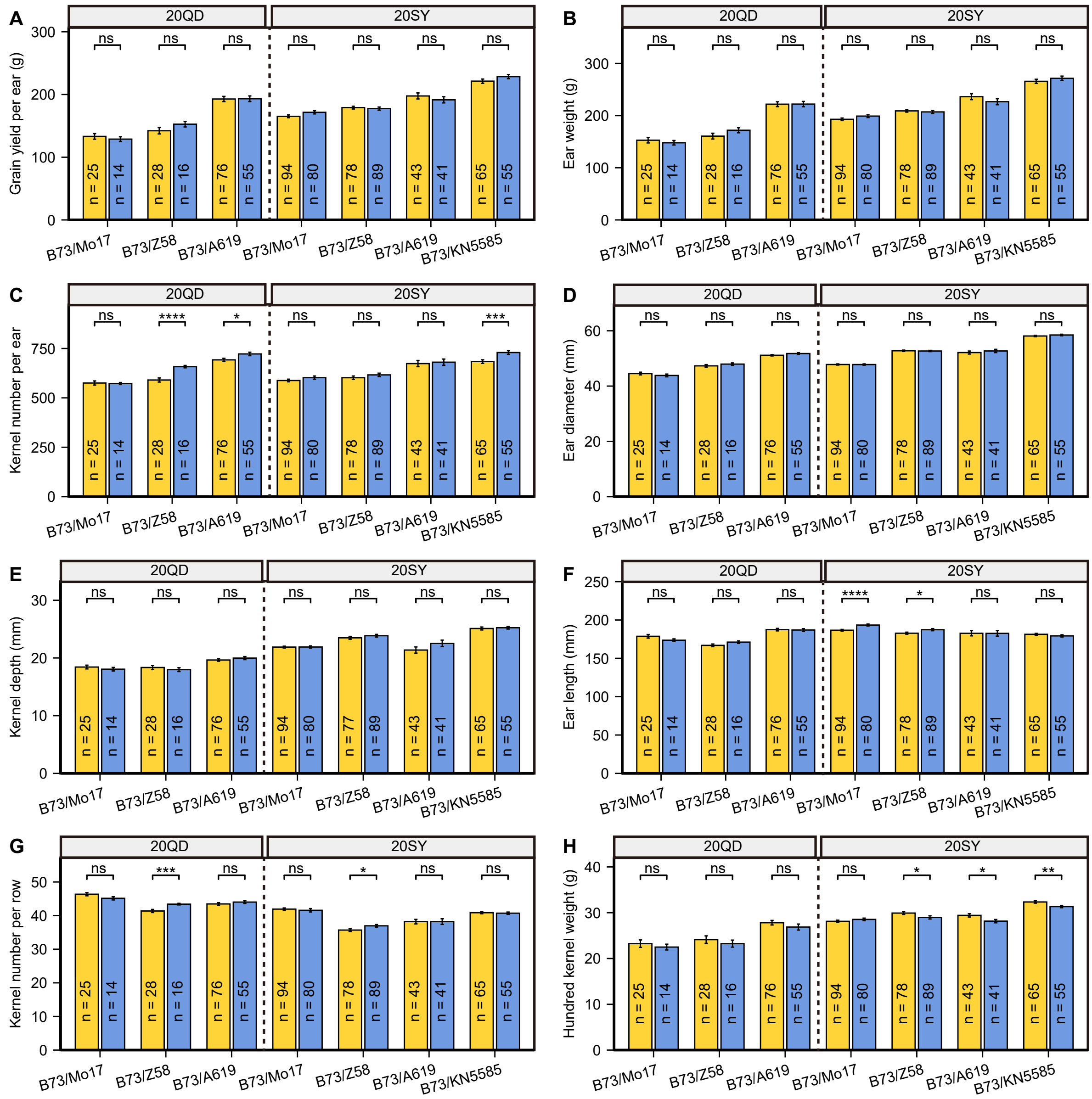

Supplementary Figure S5. Scoring of eight yield traits for *Zmcrn* heterozygotes and WT in B73/Mo17, B73/Z58, B73/A619 and B73/KN5585 hybrids at Qingdao and Sanya in 2020. Scoring of eight yield traits including grain yield per ear **A)**, ear weights **B)**, kernel numbers per ear **C)**, ear diameters **D)**, kernel depths **E)**, ear lengths **F)**, kernel numbers per row **G)**, hundred-kernel weight **H)** for segregated *Zmcrn/+* and WT in "B73/Mo17", "B73/Z58", "B73/A619" and "B73/KN5585". All yield related trait scoring were performed in two independent seasons at Qingdao and Sanya in 2020 (labelled with 20QD and 20SY). Data are presented as mean values ± s.e, * p-value ≤ 0.05, ** p-value ≤ 0.01, *** p-value ≤ 0.001, **** p-value ≤ 0.0001, with two-tailed Student's t-test. ns: not significant. The source data are listed in Supplementary Table S4.

Supplementary Figure S6

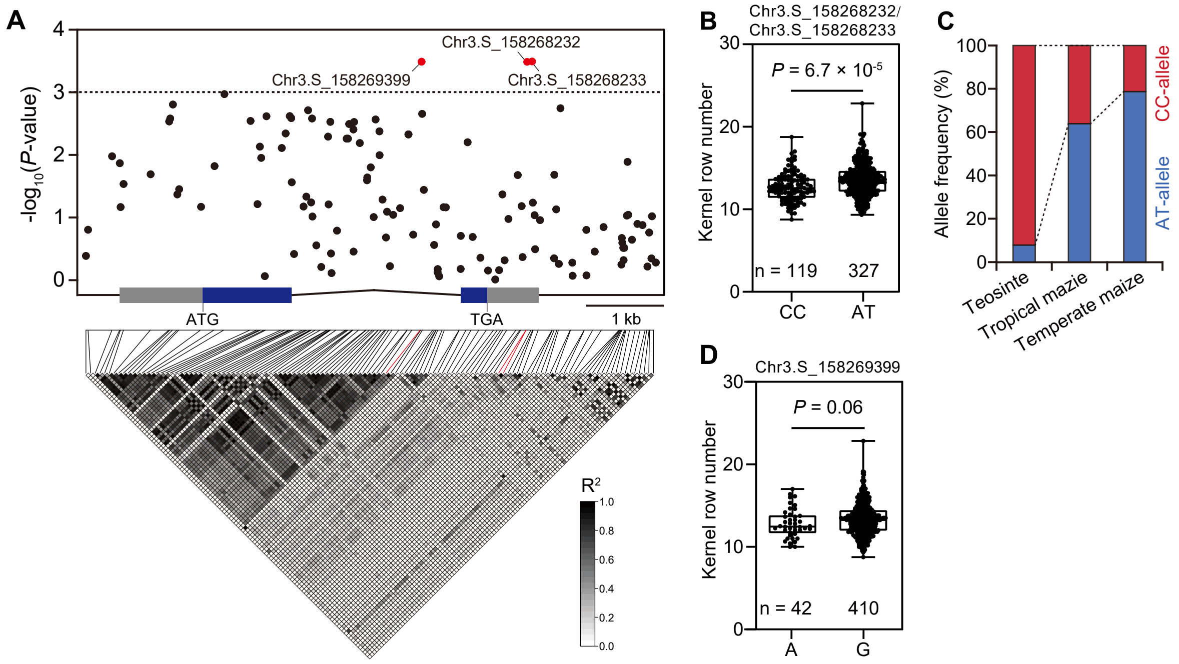

Supplementary Figure S6. Association analysis of *ZmCRN* with KRN. A) The red dots represent SNPs associate positively with KRN in diverse inbred lines. Shaded diamonds below the gene model show the SNP linkage disequilibrium by pairwise R2 values. The two SNPs at Chr3.S_158268232 and Chr3.S_158268233 were in complete linkage disequilibrium and formed two haplotypes "CC" and "AT". For the gene illustration, the blue rectangles indicate the coding sequence and the gray rectangles indicate the untranslated region (UTR). The line between the rectangles represents intron, while the lines before and after the rectangles represent the upstream and downstream regions of the gene. The red lines in the lower diagram represent the three corresponding significant SNP loci. Scale bar: 1 kb. **B)** Inbred lines carrying "AT" haplotype at Chr3.S_158268232/Chr3.S_158268233 showed significantly higher KRN than inbreds harboring "CC" haplotype at this locus. **C)** The "AT" haplotype at Chr3.S_158268232/Chr3.S_158268233 in the population rose from 8% in teosinte to 64% in tropical maize and 79% in temperate maize. 128 teosinte, 216 tropical maize, and 272 temperate maize inbred lines were analyzed. **D)** Inbreds carrying different alleles at Chr3.S_158269399 didn't exhibit a significant difference in KRN suggesting this SNP might be a false positive or weak association SNP. For **B** and **D**, The box indicates the first or third quartile with a median, whiskers further extend by ±1.5 times the interquartile range from the limits of each box. The significance is calculated using a two-tailed Student's *t*-test. n indicates the number of inbred lines.

Supplementary Figure S7

A ZmCRN

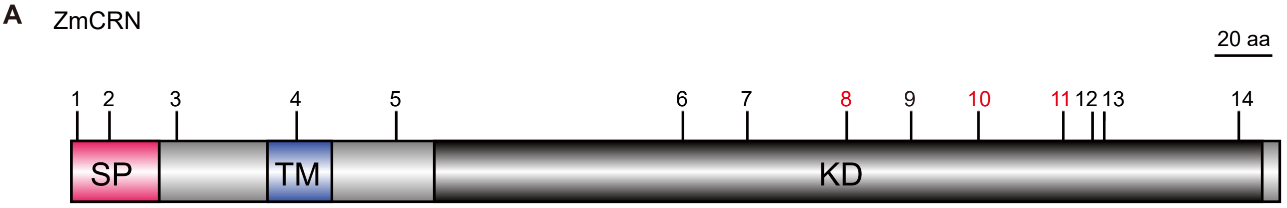

ID	Allele	Mutant ID	Effect	ID	Allele	Mutant ID	Effect
1	*Zmcrn*^{S3N}	EMS4-04dc18	aGc/aAc	8	*Zmcrn*^{S266F}	EMS4-19297b	tCc/tTc
2	*Zmcrn*^{T14I}	EMS4-04dc17	aCc/aTc	9	*Zmcrn*^{A288T}	EMS4-04dc13	Gca/Aca
3	*Zmcrn*^{E37K}	EMS4-105dbc	Gag/Aag	10	*Zmcrn*^{R311K}	EMS4-19297a	aGg/aAg
4	*Zmcrn*^{S78F}	EMS4-105dba	tCc/tTc	11	*Zmcrn*^{S340L}	EMS4-192979	tCg/tTg
5	*Zmcrn*^{P112L}	EMS4-105db9	cCg/cTg	12	*Zmcrn*^{P350S}	EMS4-105db4	Cca/Tca
6	*Zmcrn*^{D210A}	EMS4-105db7	gAc/gCc	13	*Zmcrn*^{R354W}	EMS4-04dc0e	Cgg/Tgg
7	*Zmcrn*^{G232D}	EMS4-04dc14	gGt/gAt	14	*Zmcrn*^{S400F}	EMS4-105db2	tCc/tTc

B

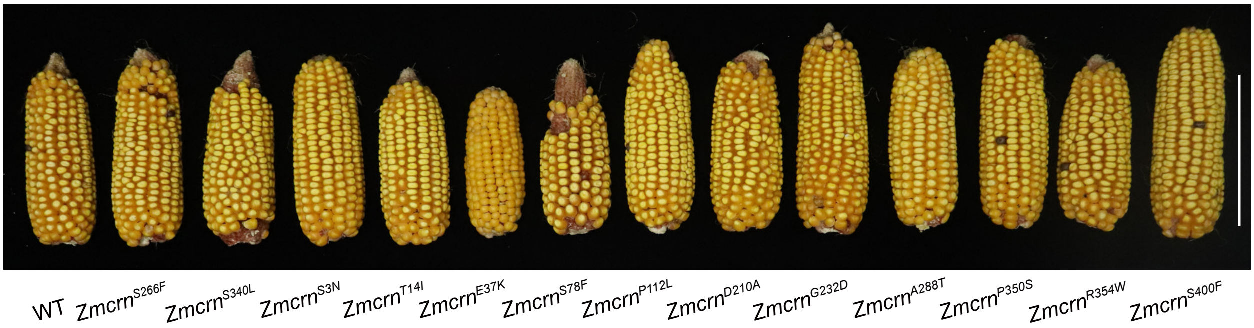

C

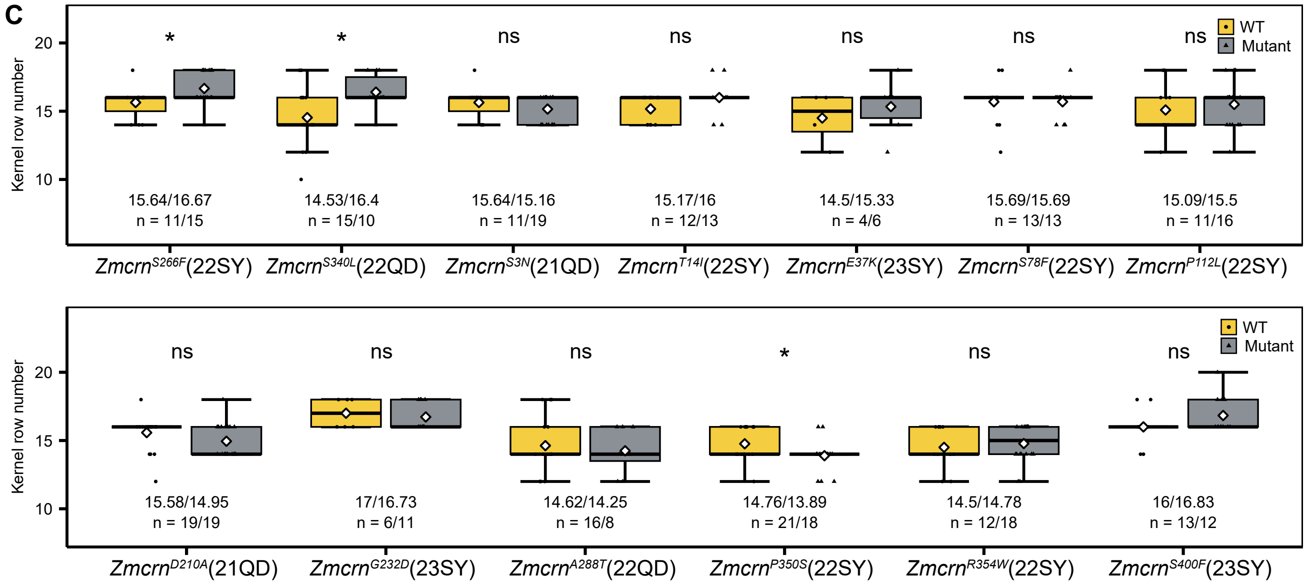

Supplementary Figure S7. Identification and ear scoring of nonsynonymous alleles of *Zmcrn*. A) Schematic of ZmCRN protein with the position of nonsynonymous alleles marked with numbers. The red text indicates weak alleles with significantly higher KRN in the mutant compared to the wild type. SP: signal peptide, TM: transmembrane domain, KD: pseudokinase domain. **B)** Representative ears for different alleles. Scale bar: 10 cm. **C)** KRN scoring for the indicated alleles at Qingdao in 2021 and 2022 (labelled with 21QD and 22QD) or Sanya in 2022 and 2023 (labelled with 22SY and 23SY). Data are presented as box plots with two-tailed Student's *t*-test. * *p*-value ≤ 0.05, ns indicates non-significant. The box indicates the first or third quartile with a median, whiskers further extend by ±1.5 times the interquartile range from the limits of each box, and the white diamond represents the mean. The mean values and the number of plants (n) used for the statistical analysis are listed. The source data and *p*-values can be found in Supplementary Table S6.

Supplementary Figure S8

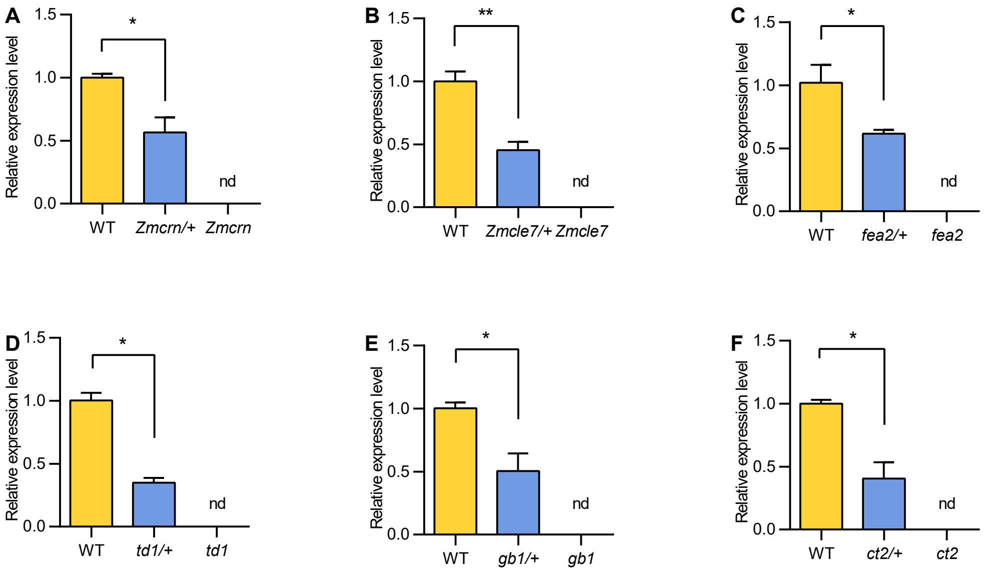

Supplementary Figure S8. Transcript levels of *FEA* genes in WT, heterozygotes and homozygotes by RT-qPCR assay. The normalized relative expression of *ZmCRN* **A)**, *ZmCLE7* **B)**, *FEA2* **C)**, *TD1* **D)**, *GB1* **E)**, CT2 **F)** in homozygotes, and heterozygotes compared to the WT siblings (normalized to *ZmACTIN*). Data are presented as mean values ± s.e with two-tailed Student's *t*-test (three biological replicates and four technical replicates). * p-value ≤ 0.05, ** p-value≤ 0.01, nd: not detectable.

Supplementary Figure S9

Gene name	Gene ID	0.6 mm ear	1.0 mm ear	1.5 mm ear	1.8 mm ear	2.0 mm ear
ZmCLE7	GRMZM2G372364	2.46	1.71	3.49	2.93	1.37
ZmCRN	GRMZM2G032132	9.34	9.53	10.89	12.30	11.63
FEA2	GRMZM2G104925	10.94	12.51	11.88	12.71	13.03
TD1	GRMZM2G300133	18.91	15.01	14.40	16.09	13.44
CT2	GRMZM2G064732	24.48	24.54	32.14	29.50	27.76
GB1	GRMZM2G045314	32.90	34.41	31.29	29.28	33.88

Supplementary Figure S9. FPKM values for different *FEA* genes in ear primordia at developmental stage. RNA seq data from (Shen et al., 2023).
