# Supplementary material for: Heterozygous fasciated ear mutations improve yield traits in inbred and hybrid maize lines
Source: Plant Physiol. 2024 Sep 11;196(4):2291–5. doi: 10.1093/plphys/kiae472 (PMC11637988; doi:10.1093/plphys/kiae472)
Supplement: kiae472_Supplementary_Data [file kiae472_supplementary_data.zip › suppMandM.pdf]

Supplementary materials and methods:

Plant materials

All *fea* mutants, including *Zmcrn* (Je et al., 2018), *Zmcle7* (Liu et al., 2021b), *fea2* (Taguchi-Shiobara et al., 2001), *td1* (Bommert et al., 2005), *ct2* (Bommert et al., 2013) and *gb1*(Wu et al., 2020) were provided by David Jackson, CSHL, New York, USA. Nonsynonymous alleles of *Zmcrn* were obtained from a gene-indexed maize (*Zea mays*) EMS mutant collection (Lu et al., 2018). B73, A619, Mo17, W22, W23 are common inbreds in the USA. RP125 (Nie et al., 2021), RP128, C7-2 and Z58(Wang et al., 2023) are elite inbreds used in Chinese breeding pipelines. KN5585 is a commonly used maize transformation receptor line in China (Liu et al., 2020).

Genotyping

Genomic DNA was extracted from approximately 100 mg young leaf tissue via a fast genomic DNA preparation method (Liu et al., 2021a). The genotyping was performed by a standard PCR program using 2 × Rapid Taq Master Mix (Vazyme, China), and the PCR products were detected by electrophoresis on 1% and 3% agarose gels based on the product size. Sanger sequencing is utilized to identify nonsynonymous alleles of *Zmcrn*. The information of primers used for genotyping is provided in Supplementary Table S7.

Evaluation of yield-related traits

The plants for yield-related trait scoring were grown in field locations at Qingdao (36°N, 120°E) during the summer season from mid-May to end of September with an average temperature from 19°C to 24.8°C. The average rainfall in Qingdao in this season is 109 mm/month. Two irrigations were applied during the seedling stage. Plants were grown at Sanya (18ºN, 109ºE) during the winter season from end of October to early March, with an average temperature around 19 to 25.3°C. The average rainfall in Sanya at this season is about 50 mm/month. Irrigation was applied every 3-4 days during the growth stage. The soil type of Qingdao is brown soil and Sanya is torrid red soil. 750 kg/ha of compound fertilizer with N≥15%, P_2O_5≥15%, and K_2O ≥15% was applied into the soil before planting for both locations. Plants were grown in low-density trial s with overall density is about 19000 plants/acre. Rows were arranged in pairs with approximately 50 cm between rows and 120 cm between pairs of rows. The plant spacing within rows was 20-25 cm. To score yield-related traits in inbred (B73) and hybrid backgrounds, heterozygous *fea* plants were crossed with B73 and other inbreds to generate segregating offspring populations of *fea* heterozygotes and wild-type. F1 seeds segregating for mutant heterozygote or WT were planted in

randomized rows with 20 plants per row. For each genotype, multiple segregating rows were planted and the WT and mutant heterozygote plants were randomly distributed in each row. During the seedling stage, the plants were numbered and genotyped by PCR. At harvest, the ears were collected and the traits are scored without knowing the genotype. The edge plants of each row were not collected to reduce the edge-effects. At last, the individuals with the trait data collected and clear genotype were subjected to data analysis. Nine yield-related traits, including kernel row number, ear length, kernel number per row, ear diameter, kernel number per row, kernel depth, hundred-kernel weight, ear weight, and grain yield per ear were measured.

Association analysis of *ZmCRN* with KRN and allele frequency analysis

Candidate-gene association mapping was carried out to identify the variants of *ZmCRN*, which were associated with KRN in a maize association population of 507 diverse inbred lines (Yang et al., 2014). High-quality SNPs and Indels were obtained from DNA whole genome re-sequencing of 507 diverse maize inbred lines with an average depth of 20 × (Chen et al.,2022). The SNP and Indels in the *ZmCRN* gene body including UTRs as well as 5 kb upstream and downstream region with the minor allele frequency (MAF) ≥ 0.05 were extracted in TASSEL version 3.0.67 (Bradbury et al., 2007). Within the investigated region, 122 high quality SNPs in the *ZmCRN* gene body and 0.5 kb upstream and 1.5 kb downstream of the gene were identified for the association analysis. However, no confident Indels were identified in these regions. All phenotypes used in this study were measured in Sanya (18ºN, 109ºE) in 2009 with two replicates and ~ten plants for each replicate. Association mapping was performed using a mixed linear model (MLM), considering population structure (Q) and relative kinship (K), in TASSEL version 3.0.67 (Shin et al., 2006; Yu et al., 2006; Yang et al., 2013). Pairwise linkage disequilibrium was calculated and then plotted using R software version 3.5.1 (Shin et al., 2006). A Bonferroni-corrected significance threshold ($p \leq 0.05/122 = 4.1E\text{-}04$) was used to identify the significant association. The SNPs information of teosinte lines are downloaded from (Chen et al., 2022).

RT-qPCR assay

The inflorescence meristems of 2-3-mm ear from WT, heterozygous and homozygous *fea* plants was used for gene expression assays. Total RNA was extracted using Direct-zol reagent (ZYMO RESEARCH) and treated with RNase-free DNase I (ZYMO RESEARCH). 1 μg total RNA was used to synthesize the first strand cDNA following the instructions of HiScript RT SuperMix for qPCR RT kit (Vazyme). A RT-qPCR analysis was subsequently performed using the 2× Universal SYBR Green Fast qPCR Mix (ABclonal) on a QuantStudio 5 System thermocycler (Thermo Fisher). The maize *ACTIN1*

(*Zm00001d010159*) gene served as an internal control to normalize the data. All *fea* mutants used in this study harbored either a Mu-insertions in exons or CRISPR induced deletions in coding region that truncated the gene function. Primers were designed to specifically amplify the WT transcript. The primers used for qRT-PCR can be found in Supplementary Table S7.

RNA-seq reads mapping and analysis

Due to the lack of *ZmCLE7* annotations in V4 B73 reference genomes , the Illumina sequencing raw data at various ear sizes (0.6 mm, 1.0 mm, 1.5 mm, 1.8 mm, 2.0 mm, 2.5 mm, and 2.0 mm) from (Shen et al., 2023) were remapped to V3 genome following the analysis process as reported in (Xu et al., 2021; Shen et al., 2023).

IM size measurement

For IM width and height measurements, 3–5-mm freshy dissected ears of heterozygous *Zmcrn* and WT siblings were imaged under a NOVEL JSZ6S stereo microscope and measured using ImageJ.

Statistical analysis

Statistical analysis was performed using two-tailed Student's *t*-test. Raw data and the specific number of plants (n) is shown in Supplementary Tables S1-S6.

Supplementary references

Bommert P, Je BI, Goldshmidt A, Jackson D (2013) The maize Gα gene COMPACT PLANT2 functions in CLAVATA signalling to control shoot meristem size. Nature **502**: 555–558

Bommert P, Lunde C, Nardmann J, Vollbrecht E, Running M, Jackson D, Hake S, Werr W (2005) *thick tassel dwarf1* encodes a putative maize ortholog of the *Arabidopsis CLAVATA1* leucine-rich repeat receptor-like kinase. Development **132**: 1235–1245

Bradbury PJ, Zhang Z, Kroon DE, Casstevens TM, Ramdoss Y, Buckler ES (2007) TASSEL: software for association mapping of complex traits in diverse samples. Bioinformatics **23**: 2633–2635

Chen L, Luo J, Jin M, Yang N, Liu X, Peng Y, Li W, Phillips A, Cameron B, Bernal JS, et al (2022) Genome sequencing reveals evidence of adaptive variation in the genus Zea. Nat Genet **54**: 1736–1745

Je BI, Xu F, Wu Q, Liu L, Meeley R, Gallagher JP, Corcilius L, Payne RJ, Bartlett ME, Jackson D (2018) The CLAVATA receptor FASCIATED EAR2 responds to distinct CLE peptides by signaling through two downstream effectors. eLife **7**: e35673

Liu H-J, Jian L, Xu J, Zhang Q, Zhang M, Jin M, Peng Y, Yan J, Han B, Liu J, et al (2020) High-Throughput CRISPR/Cas9 Mutagenesis Streamlines Trait Gene Identification in Maize. The Plant Cell **32**: 1397–1413

Liu L, Chen R, Fugina CJ, Siegel B, Jackson D (2021a) High-Throughput and Low-Cost Genotyping Method for Plant Genome Editing. Curr Protoc **1**: e100

Liu L, Gallagher J, Arevalo ED, Chen R, Skopelitis T, Wu Q, Bartlett M, Jackson D (2021b) Enhancing grain-yield-related traits by CRISPR–Cas9 promoter editing of maize CLE genes. Nat Plants **7**: 287–294

Lu X, Liu J, Ren W, Yang Q, Chai Z, Chen R, Wang L, Zhao J, Lang Z, Wang H, et al (2018) Gene-Indexed Mutations in Maize. Molecular Plant **11**: 496–504

Nie S, Wang B, Ding H, Lin H, Zhang L, Li Q, Wang Y, Zhang B, Liang A, Zheng Q, et al (2021) Genome assembly of the Chinese maize elite inbred line RP125 and its EMS mutant collection provide new resources for maize genetics research and crop improvement. Plant J **108**: 40–54

Shen X, Xiao B, Kaderbek T, Lin Z, Tan K, Wu Q, Yuan L, Lai J, Zhao H, Song W (2023) Dynamic transcriptome landscape of developing maize ear. The Plant Journal **116**: 1856–1870

Shin J-H, Blay S, McNeney B, Graham J (2006) LDheatmap: An R Function for Graphical Display of Pairwise Linkage Disequilibria Between Single Nucleotide Polymorphisms. Journal of Statistical Software **16**: 1–9

Taguchi-Shiobara F, Yuan Z, Hake S, Jackson D (2001) The *fasciated ear2* gene encodes a leucine-rich repeat receptor-like protein that regulates shoot meristem proliferation in maize. Genes Dev **15**: 2755–2766

Wang B, Hou M, Shi J, Ku L, Song W, Li C, Ning Q, Li X, Li C, Zhao B, et al (2023) De novo genome assembly and analyses of 12 founder inbred lines provide insights into maize heterosis. Nat Genet **55**: 312–323

Wu Q, Xu F, Liu L, Char SN, Ding Y, Je BI, Schmelz E, Yang B, Jackson D (2020) The maize heterotrimeric G protein β subunit controls shoot meristem development and immune responses. Proc Natl Acad Sci USA **117**: 1799–1805

Xu X, Crow M, Rice BR, Li F, Harris B, Liu L, Demesa-Arevalo E, Lu Z, Wang L, Fox N, et al (2021) Single-cell RNA sequencing of developing maize ears facilitates functional analysis and trait candidate gene discovery. Developmental Cell **56**: 557-568.e6

Yang N, Lu Y, Yang X, Huang J, Zhou Y, Ali F, Wen W, Liu J, Li J, Yan J (2014) Genome Wide Association Studies Using a New Nonparametric Model Reveal the Genetic Architecture of 17 Agronomic Traits in an Enlarged Maize Association Panel. PLOS Genetics **10**: e1004573

Yang Q, Li Z, Li W, Ku L, Wang C, Ye J, Li K, Yang N, Li Y, Zhong T, et al (2013) CACTA-like transposable element in ZmCCT attenuated photoperiod sensitivity and accelerated the postdomestication spread of maize. Proceedings of the National Academy of Sciences **110**: 16969–16974

Yu J, Pressoir G, Briggs WH, Vroh Bi I, Yamasaki M, Doebley JF, McMullen MD, Gaut BS, Nielsen DM, Holland JB, et al (2006) A unified mixed-model method for association mapping that accounts for multiple levels of relatedness. Nat Genet **38**: 203–208
